# THE RELATIONSHIP BETWEEN HEALTHCARE STEREOTYPE THREAT AND ADVANCE CARE PLANNING AMONG HISPANIC PATIENTS

**DOI:** 10.1093/geroni/igac059.1833

**Published:** 2022-12-20

**Authors:** Valeria Cardenas, Aaron Storms, Sindy Lomeli, Carin van Zyl, Susan Enguidanos

**Affiliations:** University of Southern California, San Diego , California, United States; Keck School of Medicine of USC, Los Angeles, California, United States; University of Southern California, Los Angeles, California, United States; USC, Los Angeles, California, United States; University of Southern California, Los Angeles, California, United States

## Abstract

Experiencing ethnic/racial stereotype threat, the psychological experience of confronting stereotypes in the healthcare system, has been found to negatively impact healthcare outcomes (e.g. dissatisfaction with healthcare, poorer mental and physical health, higher physician distrust). Few studies have examined healthcare stereotype threat among Hispanics, and no study has tested how it might impact advance care planning outcomes among Hispanic hospitalized patients. This study aims to examine the relationship between healthcare stereotype threat and advance care planning activities among Hispanic hospitalized patients. We analyzed secondary data collected from a feasibility study of a palliative care educational intervention among 50 Spanish-speaking, Hispanic patients, over the age of 40, who were hospitalized in a large public hospital. Fifty percent had discussed future care if they were to become seriously ill, 18% had written instructions about their care (living will or advance directive), and 32% had a durable power of attorney (proxy) for healthcare. Results of logistic regressions revealed that greater healthcare stereotype threat scores were associated with lower odds of having discussed future care or medical treatment with others (OR=0.49, p=.03). However, healthcare stereotype threat scores were not associated with having provided written instructions about future medical care (OR=0.44; p=0.07), or with having a durable power of attorney for healthcare (OR=0.89; p=0.76). Future research should investigate ways to improve the cultural sensitivity in broaching discussions of future medical treatment among seriously ill patients. Additionally, more research and attention to policy implication are needed to reduce racial stereotype threat experienced in the healthcare system.

